# A meta-analysis of based radiomics for predicting lymph node metastasis in patients with biliary tract cancers

**DOI:** 10.3389/fsurg.2022.1045295

**Published:** 2023-01-06

**Authors:** Yuhu Ma, Yanyan Lin, Jiyuan Lu, Yulong He, Qianling Shi, Haoran Liu, Jianlong Li, Baoping Zhang, Jinduo Zhang, Yong Zhang, Ping Yue, Wenbo Meng, Xun Li

**Affiliations:** ^1^ The First School of Clinical Medicine, Lanzhou University, Lanzhou, China; ^2^ Department of General Surgery, The First Hospital of Lanzhou University, Lanzhou, China; ^3^ School of Stomatology, Lanzhou University, Lanzhou, China

**Keywords:** biliary tract cancers, lymph node metastasis, radiomics, diagnosis, meta-analysis

## Abstract

**Background:**

To assess the predictive value of radiomics for preoperative lymph node metastasis (LMN) in patients with biliary tract cancers (BTCs).

**Methods:**

PubMed, Embase, Web of Science, Cochrane Library databases, and four Chinese databases [VIP, CNKI, Wanfang, and China Biomedical Literature Database (CBM)] were searched to identify relevant studies published up to February 10, 2022. Two authors independently screened all publications for eligibility. We included studies that used histopathology as a gold standard and radiomics to evaluate the diagnostic efficacy of LNM in BTCs patients. The quality of the literature was evaluated using the Radiomics Quality Score (RQS) and the Quality Assessment of Diagnostic Accuracy Studies 2 (QUADAS-2). The diagnostic odds ratio (DOR), sensitivity, specificity, positive likelihood ratio (PLR), negative likelihood ratio (NLR), and area under the receiver operating characteristic curve (AUC) were calculated to assess the predictive validity of radiomics for lymph node status in patients with BTCs. Spearman correlation coefficients were calculated, and Meta-regression and subgroup analyses were performed to assess the causes of heterogeneity.

**Results:**

Seven studies were included, with 977 patients. The pooled sensitivity, specificity and AUC were 83% [95% confidence interval (CI): 77%, 88%], 78% (95% CI: 71, 84) and 0.88 (95% CI: 0.85, 0.90), respectively. The substantive heterogeneity was observed among the included studies (*I*^2^ = 80%, 95%CI: 58,100). There was no threshold effect seen. Meta-regression showed that tumor site contributed to the heterogeneity of specificity analysis (*P* < 0.05). Imaging methods, number of patients, combined clinical factors, tumor site, model, population, and published year all played a role in the heterogeneity of the sensitivity analysis (*P* < 0.05). Subgroup analysis revealed that magnetic resonance imaging (MRI) based radiomics had a higher pooled sensitivity than contrast-computed tomography (CT), whereas the result for pooled specificity was the opposite.

**Conclusion:**

Our meta-analysis showed that radiomics provided a high level of prognostic value for preoperative LMN in BTCs patients.

## Introduction

Biliary tract cancers (BTCs) are malignant tumors derived from biliary epithelial cells, including intrahepatic and extrahepatic cholangiocarcinoma and gallbladder cancer (1–3). The global incidence is increasing (4). Radical resection is the only option to prolong survival in patients with BTCs (5). Unfortunately, less than 35% of patients are suitable for early surgery (6, 7). Recurrence after curative resection remains high, with intrahepatic cholangiocarcinoma (ICC) reaching 50%–70% (8). According to previous studies, lymph node metastasis (LMN) is the most relevant adverse prognostic factor after BTCs surgery (9). In addition, LMN is a relative contraindication for liver transplantation (10). Therefore, it is crucial to evaluate the accurate status of lymph nodes by non-introductive means, which is key for BTCs to guide treatment and determine prognosis.

Medical imaging methods play an important role in assessing the status of lymph nodes in BTCs. Conventional imaging examinations include ultrasonography, computer tomography (CT), positron emission tomography/computed tomography (PET-CT), and magnetic resonance imaging (MRI) (11, 12). Nowadays, preoperative assessment of lymph node status remains difficult. Razumilava et al. Studies have shown that CT has a sensitivity of 30%–50% in diagnosing lymph node status (7). There is no consensus on the evaluation of the preoperative lymph node status of BTCs by current detection methods (13, 14). A label non-invasive detection method called radiomics has recently been utilized to predict chemotherapy response, lymph node metastasis, and tumor classification (15). it extracts high-dimensional radiomics information from medical images and combines machine learning algorithms for clinical decision-making (16, 17). A systematic review and meta-analysis by Huang et al. found that radiomics might be an effective tool for assessing preoperative microvascular invasion in hepatocellular carcinoma (17). Several published studies have employed a radiomics model to predict LNM in BTCs (18–20). Due to differences in imaging modality, study methodology, sample size imaging modalities, research methods, sample size and so on, the reported diagnostic efficiency ranged from 68% to 98% in the above studies. Therefore, the performance of radiomics for preoperative LMN identification in clinical practice remains uncertain.

The purpose of this meta-analysis was to determine the diagnostic efficacy of radiomics for preoperative LMN prediction in patients with BTCs.

## Materials and methods

This meta-analysis was conducted following the Preferred Reporting Items for Systematic Reviews and Meta-Analyses (PRISMA) statement recommended by the Cochrane Collaboration (21). This study was prospectively registered in PROSPERO (CRD42022333874).

### Literature search

Two authors (YM and YH) independently searched PubMed, Embase, Web of Science, Cochrane Library, and four Chinese Databases [VIP, CNKI, Wanfang, and Chinese BioMedical Literature Databases (CBM)] to determine the studies published as of February 10, 2022. The search formula was as follows: [(lymph node metastasis) OR (lymph node) OR(LMN)] and [(Biliary Tract Cancer) OR (intrahepatic cholangiocarcinoma) OR (extrahepatic cholangiocarcinoma) OR (ICC) OR (ECC)] and [(radiomics) OR (machine learning) OR (deep learning) OR (artificial intelligence) OR (texture)]. After eliminating duplicate articles, the titles and abstracts of all remaining articles were reviewed. When it was ambiguous whether the article was included merely by title and abstract, the entire publication was downloaded and reviewed. All studies were independently screened by two authors (YM and YH). Discuss the inclusion issues if there were any discrepancies. In order to find other relevant publications, we also carefully went through the reference lists for each important study that we had already identified as well as earlier systematic reviews.

### Selection criterion

Inclusion criteria were as follows: (1) diagnosis of BTCs by pathologic criteria; (2) determination of LMN by pathologic diagnosis; (3) CT, MRI, PET-CT, or ultrasonography were performed before surgical resection, liver transplantation, or other treatments; (4) imaging analysis based on radiomics.

Exclusion criteria are as follows: (1) having received any treatment (radiotherapy, chemotherapy, or immunotherapy) before the examination; (2) Patients who received palliative surgery without lymph node resection; (3) Reviews, editorials, letters, and animal articles are excluded.

### Quality assessment

The Radiomics Quality Score (RQS) and Quality Assessment of Diagnostic Accuracy Studies 2 (QUADAS-2) were used by the two authors (YM and YH) to evaluate the methodological quality and risk of bias of the chosen studies independently, respectively (22, 23).

### Data extraction

The data extraction and quality evaluation of the retrieved research are independently completed by the two authors. We extracted data about patient characteristics, imaging methods, and research characteristics from each selected study. Patient characteristics included the total number of subjects, the number of subjects with LMN and no-LMN, sensitivity, and specificity. The number of true positive (TP), true negative (TN), false positive (FP), and false negative (FN) was calculated according to the number of LMN, non-LMN, sensitivity, and specificity reported in each included study. The reference formula was as follows: sensitivity = TP/(TP+FN), specificity = TN/(FP+TN). The studies that provide a two-by-two contingency table or sufficient data to reconstruct such a table are eligible for analysis. The best model presented in the study was included in our meta-analysis when there were two or more prediction models based on the same cohort of patients in one study.

### Statistical analysis

Statistical analysis was performed using Stata software (version 16.0) and Review Manager software (version 5.3). The sensitivity, specificity, positive likelihood ratio (PLR), negative likelihood ratio (NLR), and diagnostic odds ratio (DOR) with their corresponding 95% confidence intervals (CI) were calculated. A summary receiver operating characteristic (sROC) curve was plotted and the area under the curve (AUC) was calculated to demonstrate the diagnostic value of the joint studies (24). AUC was 0.5–0.7, 0.7–0.9, and > 0.9, indicating low, medium, and high diagnostic power, respectively.

We drew forest plots to show the variation among studies and to detect heterogeneity for the pooled sensitivity and specificity. The threshold effect resulting in heterogeneity was assessed using the spearman correlation coefficient. If *P *> 0.05, there was no threshold effect. The heterogeneity caused by the non-threshold effect was measured by Cochrane's Q-test and inconsistency index *I*^2^. When *P *< 0.05, the difference was considered significant, and *I*^2 ^≥ 50% was considered moderate to high heterogeneity among studies (25). Meta-regression and subgroup analysis were used to study the potential sources of heterogeneity. We conducted a univariate meta-regression analysis of some related covariates, including the tumor site (ICC or no-ICC), combined clinical factors (yes or no), imaging methods (MRI or CT), number of patients (≥ 150 or < 150), QUADAS-2 applicability risk (no or high risk), model (logistic regression or machine learning), population (single or multicenter), number of radiomics features (≥ 300 or < 300), and published year (before 2020 or after 2020). Additionally, a sensitivity analysis was carried out by removing one study at a time to assess the effect of a single study on the overall estimation. Deeks' funnel plot was used to check publication bias (26).

### Clinical utility

A Fagan plot was calculated to assess clinical utility by indicating the post-test probability (*P*-post) of LNM when pretest probabilities (*P*-pre, suspicion of LNM) were provided (27).

## Results

### Literature search

**Figure 1:**
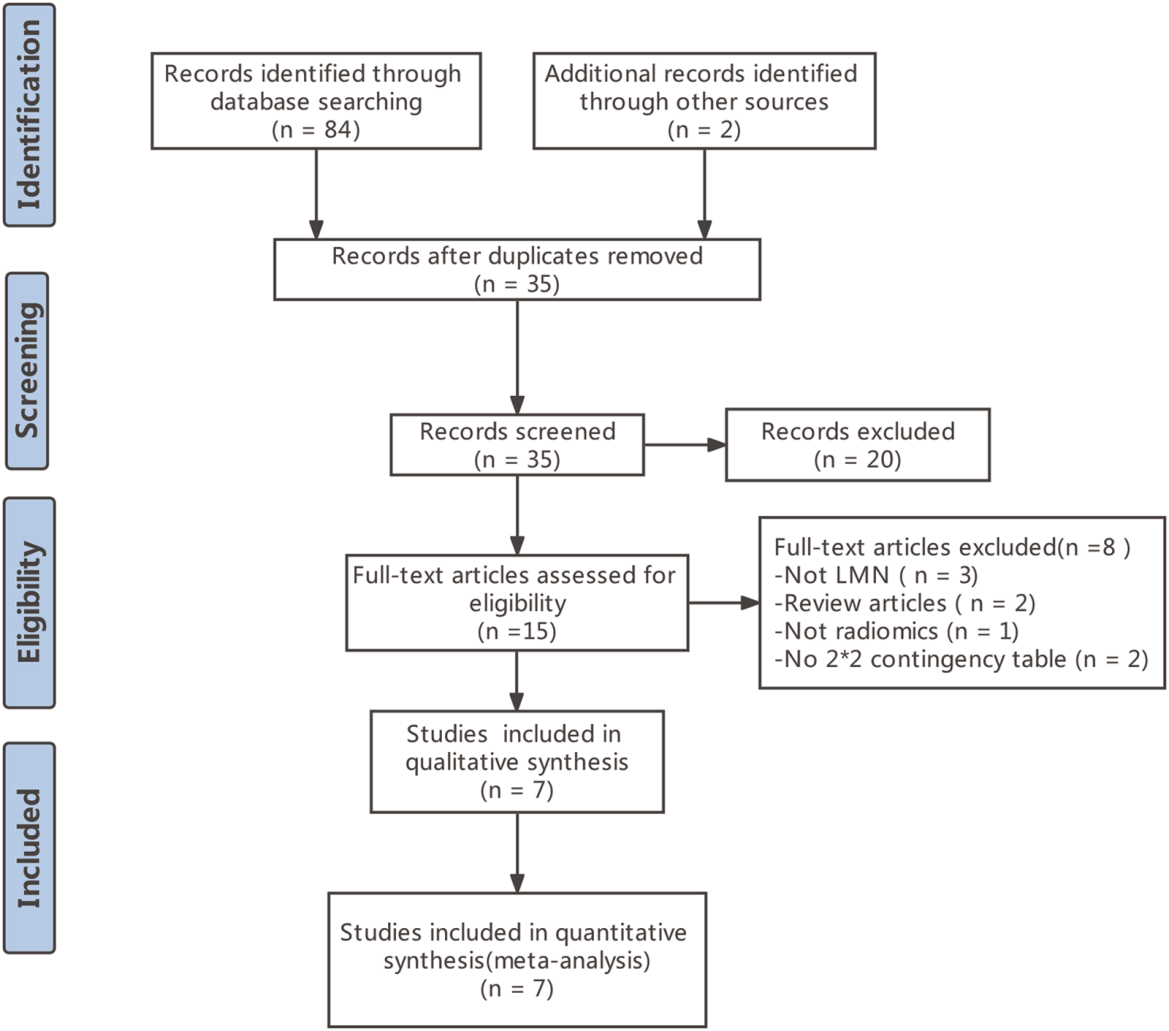
The PRISMA flowchart of the selection procedure.

The literature search and study selection was shown in Figure 1. The included studies were published between 2018 and 2021 (four contract-CT based on radiomics studies (18, 28, 29) and three MRI based on radiomics studies (19, 30–32) were included in the meta-analysis). A total of 977 BTCs patients were included. Of those, 554 patients (56.8%) had a pathological diagnosis of no-LMN, while 423 patients (43.2%) had a pathological diagnosis of LMN. The baseline characteristics of the included studies was showed in Table 1.

**Table 1:** The baseline characteristics of the included 7 studies.

Study ID	Year	Tumor type	Imaging methods	Sequence	Study design	Population	ROI	Model	Feature number	Numbers of patients	LMN	No-LMN	TP	FP	FN	TN	Combine Clinical Factors (Yes/No)
Ji et al.	2019	ICC	Contrast-CT	Arterial phase	retrospective	Single center	Tumor parenchyma	LASSO and LR	105	103	45	48	39	15	6	43	Yes
Yang et al.	2020	ECC	MRI	T1WI, T2WI, DWI	retrospective	Single center	Tumor parenchyma	MI and RF	300	100	73	27	58	5	15	22	No
Xu et al.	2019	ICC	MRI	T1WI	retrospective	Single center	Tumor parenchyma	mRMR and SVM	491	106	47	59	42	25	5	34	Yes
Liu et al.	2021	GBC	Contrast-CT	Venous phase	retrospective	Multicenter	Tumor parenchyma	LASSO and LR	293	209	84	125	73	28	11	97	Yes
Ji et al.	2018	BTCs	Contrast-CT	Venous phase	retrospective	Single center	Tumor parenchyma	LASSO and LR	93	177	35	35	16	7	19	28	Yes
Yao et al.	2020	ECC	MRI	T1WI, T2WI, DWI and ADC	retrospective	Single center	Approximately 1–2 mm from the edge of the tumor	PSO and SVM	120	110	79	31	68	6	11	25	No
Huang et al.	2019	ICC	Contrast-CT	Complete sequence	retrospective	Single center	NA	RF and LR	832	172	51	121	45	21	6	100	Yes

BTCs, biliary tract cancers; ECC, extrahepatic cholangiocarcinoma; ICC, intrahepatic cholangiocarcinoma; ROI: region of interest; GBC, gallbladder cancer; LASSO, least absolute shrinkage, and selection operator; SVM, support vector machine; mRMR, minimum redundancy maximum relevance; RF, random forest; LR, logistic regression.

### Study evaluation

A detailed report of the RQS project scores was shown in Table 2. The RQS included in the study ranged from 11 to 20 points. The publication with the highest percentage of RQS was 56.0%. Indicating excellent reproducibility across readers, the interclass correlation coefficient (ICC) among separate readers rating publications was 0.972 (95% CI: 0.854–0.997, *P *< 0.001). The RQS scores assessed by both readers were presented in Supplementary materials.

**Table 2:** RQS elements and the mean rating of our eligible studies.

RQS scoring item	Interpretation	Average score
Image Protocol	+ 1 for well documented protocols, + 1 for publicly available protocols	2.00
Multiple Segmentations	+ 1 if segmented multiple times (different physicians, algorithms, or perturbation of regions of interest)	0.64
Phantom Study	+ 1 if texture phantoms were used for feature robustness assessment	0.00
Multiple Time Points	+ 1 multiple time points for feature robustness assessment	0.00
Feature Reduction	−3 if nothing, + 3 if either feature reduction or correction for multiple testing	3.00
Non Radiomics	+ 1 if multivariable analysis with non-radiomics features	0.71
Biological Correlates	+ 1 if present	0.00
Cut-off	+ 1 if cutoff either pre-defined or at median or continuous risk variable reported	0.57
Discrimination and Resampling	+ 1 for discrimination statistic and statistical significance, + 1 if resampling applied	1.50
Calibration	+ 1 for calibration statistic and statistical significance, + 1 if resampling applied	1.21
Prospective	+ 7 for prospective validation within a registered study	0.00
Validation	−5 if no validation/+2 for internal validation/+3 for external validation/+4 two external validation datasets or validation of previously published signature/+5 validation on ≥3 datasets from >1 institute	2.14
Gold Standard	+ 2 for comparison to gold standard	1.42
Clinical Utility	+ 2 for reporting potential clinical utility	1.14
Cost-effectiveness	+ 1 for cost-effectiveness analysis	0.00
Open Science	+ 1 for open-source scans, + 1 for open-source segmentations, + 1 for open-source code, + 1 open-source representative segmentations and features	2.14

**Figure 2:**
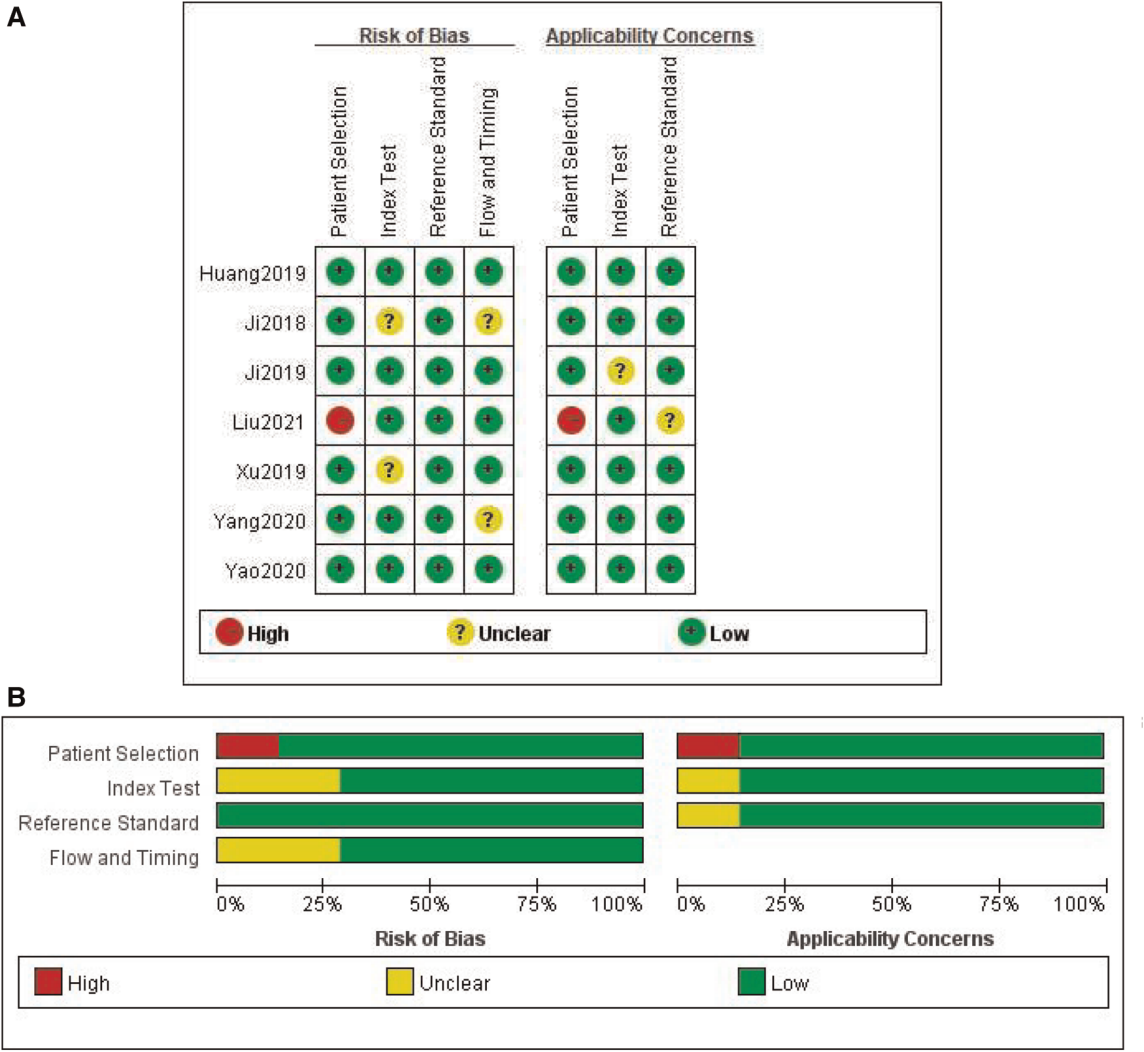
Methodological quality assessment of the included studies based on the QUADAS-2 scale. (**A**) Individual studies, (**B**) summary.

**Figure 3:**
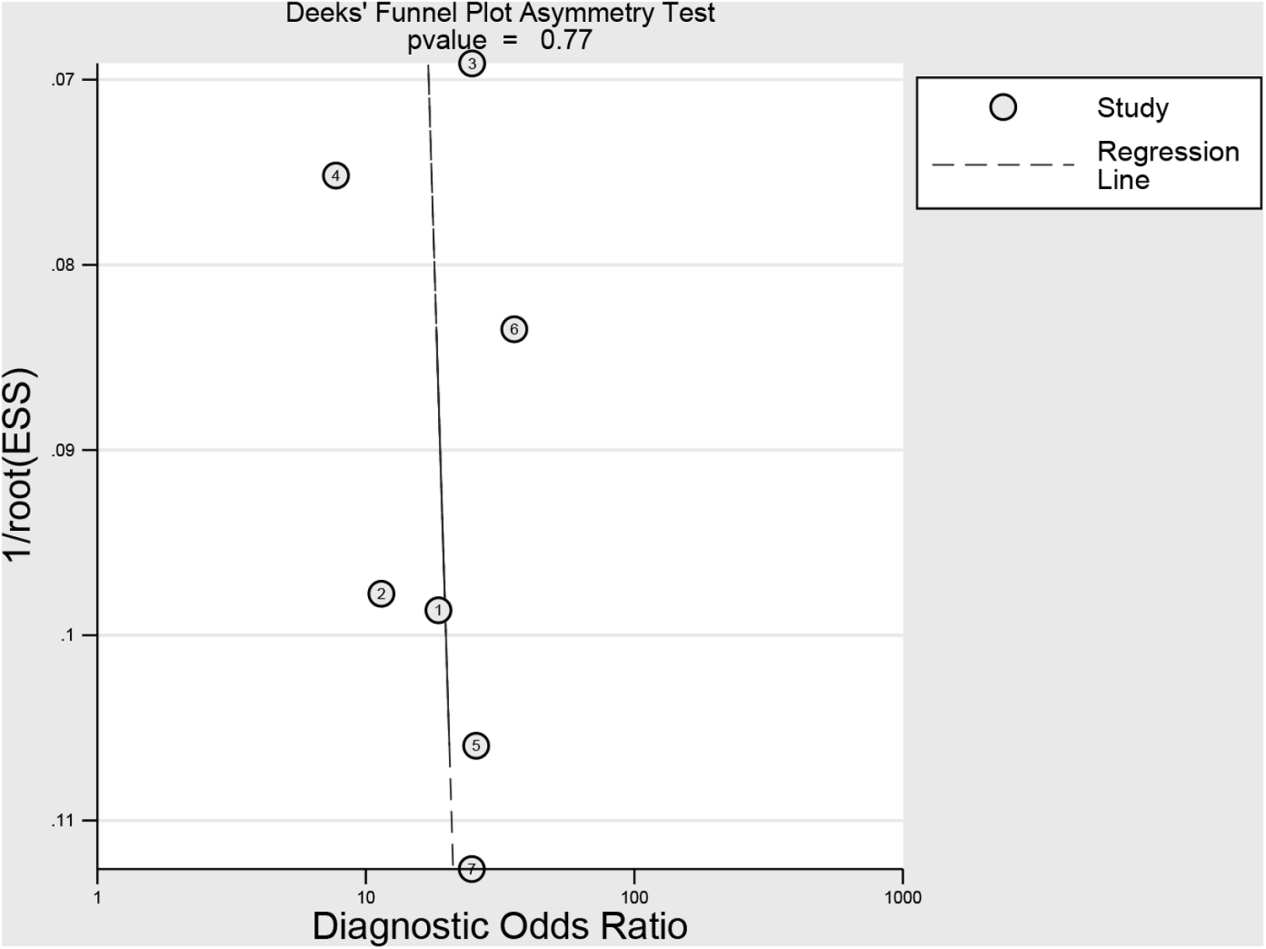
Deeks’ funnel plot. ESS, effective sample size.

The methodological quality of the studies according to the QUADAS-2 assessment was illustrated in Figure 2. Two studies obtained an unclear risk of bias in the index testing (28, 30). Uncertain risk of bias was found in both the flow and time domains in two studies (19, 28). There were relatively few concerns about the applicability of the three domains (patient selection, index test, and reference criteria). No significant publication bias risk was detected by Deeks' funnel plot analysis (Figure 3; *P* = 0.77).

### Diagnostic accuracy of radiomics

**Figure 4:**
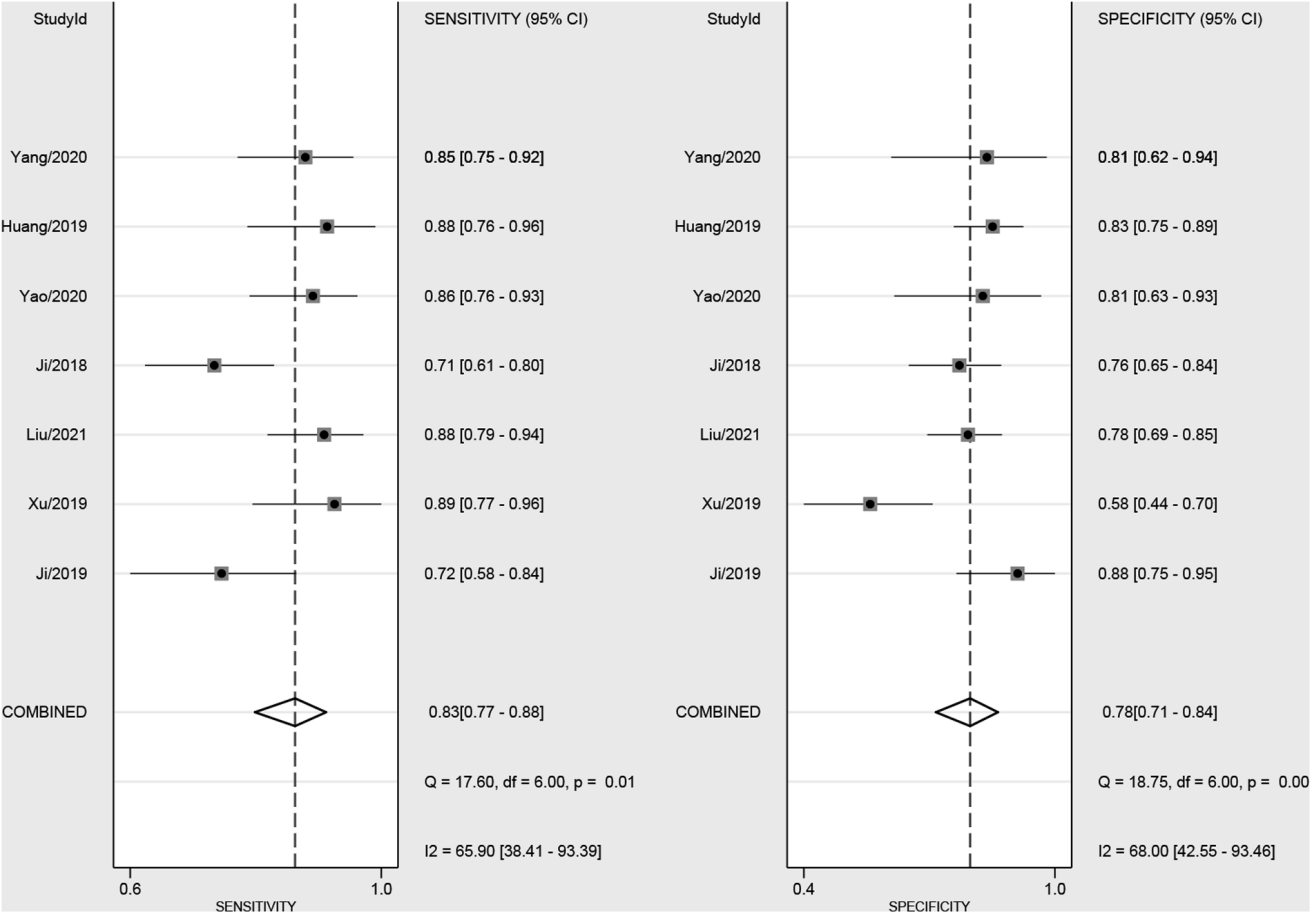
Forest plot of sensitivity and specificity based on radiomics for preoperative prediction of LMN in BTCs.

**Figure 5:**
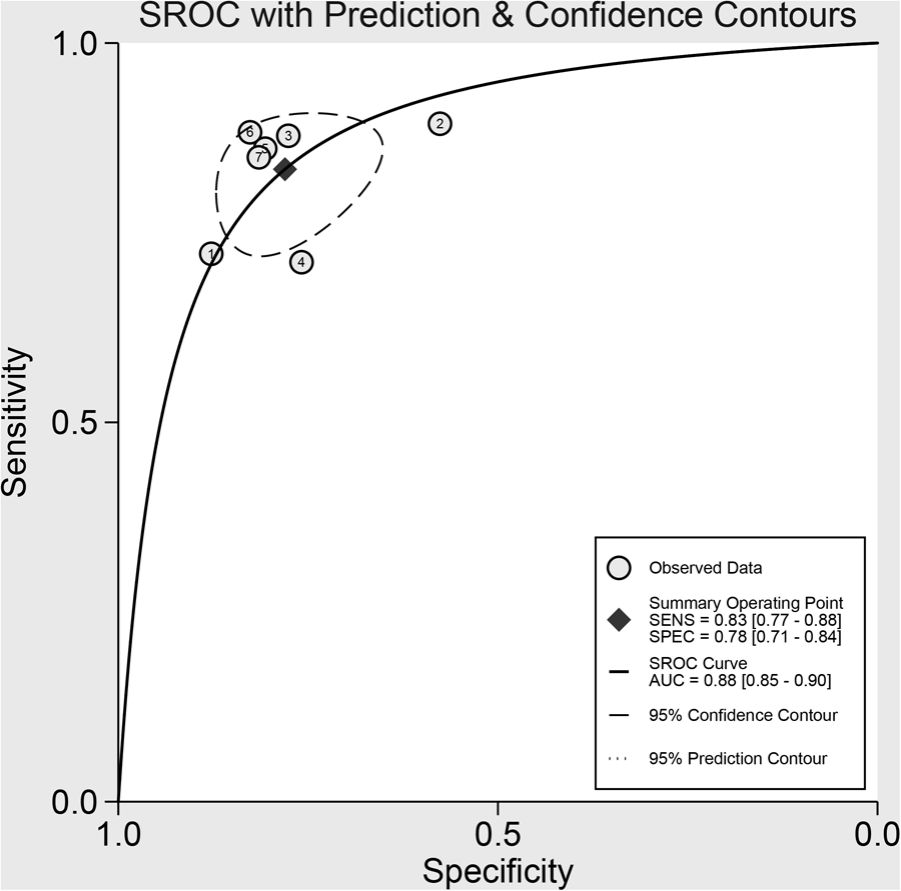
Summary receiver operating characteristic curves (sROC) based on radiomics for preoperative prediction of LMN in BTCs.

The pooled sensitivity and specificity were 83% (77%, 88%) and 78% (71%, 84%), respectively, based on radiomics assessment of lymph node status in each patient. AUC, DOR, PLR and NLR were 0.88 (0.85, 0.90), 17.82 (11.42, 27.80), 3.80 (2.88, 5.00) and 0.21 (0.16, 0.29), respectively. Figure 4 shows the forest plots for sensitivity and specificity, while Figure 5 shows the sROC curve.

### Heterogeneity assessment

The Spearman correlation coefficient for the threshold effect was found to be −0.67 and *P* = 0.45, indicating that there was no threshold effect. There was considerable heterogeneity among the studies (overall *I*² = 80%; 95%CI: 58.00,100.00; *P* = 0.003). The forest plots indicated high heterogeneity with *I^2^* values > 50% for sensitivity (*I*² = 65.90%; 95% CI: 38.41, 93.39; *P *= 0.01) and specificity (*I²* = 68.00%; 95% CI: 42.55, 93.46; *P* < 0.01).

### Meta-regression

A univariate meta-regression analysis was used to determine the sources of heterogeneity. The outcomes of subgroup analysis and univariate meta-regression were displayed in Table 3. The results showed that the sources of heterogeneity in the sensitivity analysis, in addition to QUADAS, included number of radiomics features, tumor site, imaging methods, number of patients, combined clinical factors, model, population, and publication year (*P* < 0.05). Additionally, tumor site contributed to the heterogeneity in the specificity analysis (*P* < 0.05).

**Table 3:** Univariable meta-regression and subgroup analyses.

Subgroup	Category	No. of Studies	Sensitivity (95% CI)	*P* value	Specificity (95% CI)	*P* value
Tumor site	ICC	3	0.84 (0.76–0.93)	0.00	0.78 (0.68–0.87)	0.03
	No-ICC	4	0.83 (0.76–0.90)		0.78 (0.70–0.85)	
Combine clinical factors	Yes	5	0.82 (0.76–0.89)	0.00	0.77 (0.70–0.85)	0.06
	No	2	0.86 (0.77–0.94)		0.81 (0.69–0.94)	
Imaging methods	CT	4	0.80 (0.73–0.87)	0.00	0.81 (0.75–0.86)	0.18
	MRI	3	0.87 (0.81–0.93)		0.71 (0.60–0.82)	
No. of participants	≥150	3	0.82 (0.74–0.91)	0.01	0.79 (0.71–0.87)	0.06
	<150	4	0.84 (0.77–0.91)		0.77 (0.67–0.86)	
QUADAS	High risk	1	0.88 (0.78–0.98)	0.33	0.78 (0.63–0.93)	0.18
	No high risk	6	0.82 (0.77–0.88)		0.78 (0.71–0.85)	
Model	LR	4	0.80 (0.74–0.87)	0.00	0.80 (0.73–0.88)	0.11
	ML	3	0.87 (0.81–0.94)		0.75 (0.65–0.85)	
Population	Single center	6	0.82 (0.77–0.88)	0.02	0.78 (0.71–0.85)	0.19
	Multicenter center	1	0.88 (0.78–0.98)		0.78 (0.63–0.93)	
No. of features	≥300	3	0.88 (0.81–0.94)	0.00	0.75 (0.65–0.85)	0.11
	<300	4	0.80 (0.74–0.87)		0.80 (0.73–0.88)	
Published year	After 2020	3	0.86 (0.81–0.92)	0.03	0.80 (0.70–0.89)	0.08
	Before 2020	4	0.80 (0.72–0.87)		0.77 (0.69–0.85)	

ICC, intrahepatic cholangiocarcinoma; MRI, magnetic resonance imaging; CT, computed tomography; QUADAS, quality assessment of diagnostic accuracy studies; LR, logistic regression; ML, machine learning.

### Subgroup analysis

In terms of tumor site, the sensitivity (84%; 95% CI: 76, 93 vs. 83%; 95% CI: 76, 90) and specificity (78%; 95% CI: 68, 87 vs. 78%; 95% CI: 70, 85) were basically equivalent among studies with ICC studies (*n* = 3) and no-ICC studies (*n* = 4). In comparison to radiomics combined with clinical risk factors, radiomics alone had better sensitivity (86%; 95% CI: 77, 94 vs. 82%; 95% CI: 76, 89) and specificity (81%; 95% CI: 69, 94 vs. 77%; 95%; CI: 70, 85). Moreover, the pooled sensitivity (86%; 95% CI: 81, 92 vs. 80%; 95% CI: 72, 87) and specificity (80%; 95% CI: 70, 89 vs. 77%; 95% CI: 69, 85) of studies published after 2020 (*n* = 3) was relatively high than that of earlier studies (*n* = 4). Regardless of the QUADAS risk, the specificity (78%;95% CI: 63, 93 vs. 78%; 95% CI: 71, 85) was roughly same for both. Furthermore, QUADAS high-risk studies (*n* = 3; 88%; 95% CI: 78, 98) had a marginally greater sensitivity than no high-risk studies (*n* = 4; 82%; 95% CI: 77, 88).

Among different imaging methods, MRI (*n* = 3) had higher sensitivity (87%; 95% CI: 81, 93 vs. 80%; 95% CI: 73, 87), but the specificity of contract-CT (81%; 95% CI: 75, 86) better than MRI (71%; 95% CI: 60, 82). In addition, multicenter center studies (*n* = 1) exhibited greater sensitivity (88%; 95% CI: 78, 98 vs. 82%; 95% CI: 77, 88) than s single-center studies (*n* = 6). The pooled sensitivity for more radiomics features (*n* = 3) was higher (88%; 95% CI: 81, 94 vs. 80%; 95% CI: 74, 87), whereas the trend for pooled specificity was the opposite (75%; 95% CI: 65, 85 vs. 80%; 95% CI: 73, 88). Similarly, studies with 150 patients or fewer (*n* = 4) had a greater pooled sensitivity (84%; 95% CI: 77, 91) but a lower specificity (77%; 95% CI: 67, 86) than studies with more than 150 patients (*n* = 3; sensitivity, 82%; 95% CI: 74, 91; specificity, 79%; 95% CI: 71, 87). For modeling methods, machine learning (*n* = 3) approach had exhibited better sensitivity (87%; 95% CI: 81, 93)] than logistic regression (*n* = 4; 79%; 95% CI: 73, 86) and lower specificity [72%; 95% CI: 60, 85) than logistic regression (79%; 95% CI: 71, 87).

### Sensitivity analyses

No significant changes were observed when each included study was eliminated from the analysis one by one. The results of sensitivity analyses for each study are shown in Table 4.

**Table 4:** The results of sensitivity analyses for each study.

Study ID	sROC	Sensitivity (95% CI)	Specificity (95% CI)	PLR (95% CI)	NLR (95% CI)	DOR (95% CI)
Ji et al.	0.88 (0.85–0.90)	0.85 (0.79–0.89)	0.76 (0.69–0.82)	3.58 (2.72–4.72)	0.20 (0.11–0.28)	17.88 (10.71–29.87)
Yang et al.	0.87 (0.84–0.90)	0.83 (0.76–0.89)	0.78 (0.70–0.84)	3.74 (2.77–5.03)	0.22 (0.15–0.31)	17.24 (10.61–28.01)
Xu et al.	0.82 (0.79–0.85)	0.82 (0.76–0.87)	0.80 (0.76–0.84)	4.16 (3.40–5.10)	0.22 (0.16–0.30)	18.93 (11.91–30.09)
Liu et al.	0.87 (0.84–0.90)	0.82 (0.75–0.88)	0.78 (0.69–0.85)	3.77 (2.69–5.27)	0.23 (0.16–0.31)	16.74 (10.03–27.95)
Ji et al.	0.90 (0.87–0.92)	0.85 (0.80–0.90)	0.79 (0.70–0.86)	4.07 (2.90–5.73)	0.18 (0.14–0.24)	22.08 (14.52–33.57)
Yao et al.	0.87 (0.84–0.90)	0.83 (0.76–0.88)	0.78 (0.70–0.84)	3.74 (2.77–5.06)	0.22 (0.16–0.31)	17.02 (10.48–27.63)
Huang et al.	0.87 (0.83–0.89)	0.83 (0.76–0.88)	0.77 (0.69–0.83)	3.56 (2.65–4.77)	0.23 (0.16–0.31)	15.79 (10.11–24.67)

sROC, Summary receiver operating characteristic curves; PLR, positive likelihood ratio; NLR, negative likelihood ratio; DOR, diagnostic odds ratio.

### Clinical utility

**Figure 6:**
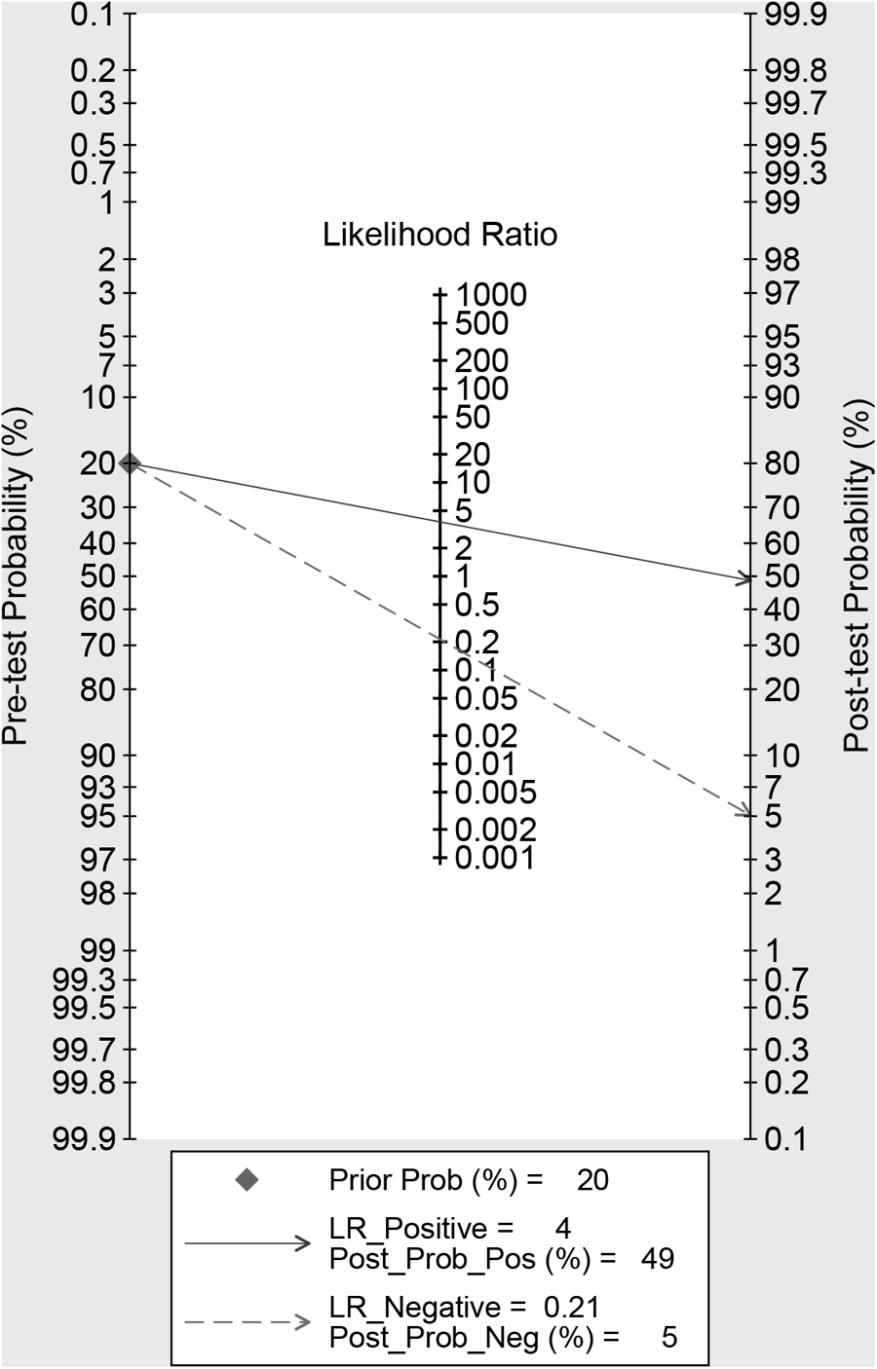
Fagan plots for assessing the clinical utility.

Using radiomics studies would increase the posttest probability to 49 from 20% with a PLR of 4 when the pretest was positive and would reduce the posttest probability to 5% with an NLR of 0.21 when the pretest was negative (Figure 6).

## Discussion

The seven studies were included in the meta-analysis. The diagnostic efficacy of radiomics for preoperative LMN in BTCs patients was judged by combining diagnostic effect size and fitting the sROC curve. Analyze the heterogeneity of the studies and their sources, and identify factors that may affect the results. Finally, through sensitivity analysis, publication bias and clinical application value were detected to evaluate the credibility of this meta-analysis.

Our meta-analysis showed high sensitivity (83%; 95% CI: 77%, 88%), specificity (78%; 95% CI: 71, 84) and AUC (0.88; 95% CI: 0.85, 0.90). In addition, the likelihood ratio and post-test probability indicate that the post-test probability increases from 20% to 49% when the current test is positive and the PLR is 4; when the current measurement is negative and the NLR is 0.21, the post-test probability is reduced to 5%. This further indicates that radiomics is helpful to improve the accuracy of predicting LMN in BTCs patients. All things considered, radiomics can assist us in possible resolution treatment protocols for BTCs LMN patients before surgery, increase the survival rate of BTCs patients, and decrease the probability of recurrence.

Currently, using visual observation and interpretation of medical images to evaluate lymph node metastasis in BTCs is still challenging (33). Radiomics, as a personalized assessment tool, has proved to be a promising non-invasive tumor lymph node evaluation to overcome the limitations of visual assessment of lymph node images by imaging physicians (34). Our findings demonstrate that radiomics can increase to 83% the sensitivity of preoperative BTCs lymph node metastatic evaluation. The radiomics method, which offers crucial supplementary information on imaging phenotypes and may contain a wealth of data, maybe the cause. This includes texture features that reflect the pattern or spatial distribution of voxel intensity in the region of interest (ROI), which are connected to tumor heterogeneity (35, 36). Wavelet features can also offer multi-frequency information to measure tumor heterogeneity and raise diagnostic accuracy (35, 37, 38). Therefore, we have reason to believe that radiomics, which is used by professional radiologists for ROI segmentation and high-throughput feature extraction for disease classification and prognosis, may be more suitable for the preoperative evaluation of LMN in patients with BTCs.

Although there was no discernible threshold effect, there was overall significant heterogeneity between studies (*I*² = 80%; 95%CI: 58, 100; *P* = 0.003). We conducted meta-regression to detect the sources of heterogeneity. Due to the limited number of included studies, only univariable meta-regression analysis was performed. The results revealed that factors such as the tumor site, imaging methods, number of patients, combined clinical factors, number of radiomics features, model, population, and published year all contributed to the heterogeneity of sensitivity analysis. In addition, the methodologies utilized in each of the included studies varied, contributing to the heterogeneity in a way that made it impossible to identify all of its sources.

We used key factors for subgroup analysis. In the study design subgroup analysis, the imaging methods results showed that the pooled sensitivity of MRI imaging is better than that of contract-CT, which is similar to the previous research results (28, 39, 40). From the number of features and modeling methods of the included studies, the extraction of more radiomics features could improve the pooled sensitivity, and the possible reason texture features could improve the accuracy of the model. However, numerous features had an impact on feature selection and model robustness, so its specificity was lower. Machine learning still needs to be investigated in terms of feature selection and model generalization potential because it can increase sensitivity but decrease specificity.

Studies have demonstrated that in patients with BTCs, the CA199 and CT lymph node status are independent predictors of LMN (18, 36). Our meta-analysis showed that combined clinical factors did not improve the diagnostic ability of radiomics. Therefore, combining clinical features in radiomics to improve the diagnostic accuracy of LNM needs further exploration. The diagnostic sensitivity of ICC was slightly higher than no-ICC, but the overall diagnostic efficacy had no significant difference. It is reasonable to think that more multicenter studies in the future will increase the predictive performance of MLN in patients with BTCs because its pooled sensitivity was greater than that of single-center studies. There is only one high-risk trial, however, there is no obvious heterogeneity in the sensitivity or specificity of the QUADAS-2 score. The ability to identify LMN in the future study will be improved by controlling study quality to lessen bias.

Despite radiomics' strong capacity to predict, the quality of the included research as a whole range (RQS range from 11 points to 20 points). There is no cost-benefit analysis or prospective design. One study only received external validation. The QUADAS-2 data quality assessment revealed some additional issues. For example, index testing results from two studies showed uncertain bias risks.

Our meta-analysis that used radiomics to predict the lymph node status of preoperative BTCs offered two advantages. First of all, this study, which was the first meta-analysis to assess the diagnostic efficacy of preoperative prediction of lymph node status in BTCs patients by radiomics evaluation method, involved seven studies and 977 BTCs patients. Second, we used subgroup analysis to evaluate the effects of different factors on the heterogeneity of the studies, providing a guide for upcoming radiomics research and clinical evaluation.

There are some limitations to our study. First, there are few qualified radiomics studies, and different medical centers use various inspection equipment. As a result, research methods vary from study to study, and imaging methods, ROI, feature extraction, and modeling methods provide many options. Second, different imaging methods, number of patients, and tumor sites may lead to heterogeneity. Therefore, we use regression analysis to identify the sources of heterogeneity. Finally, while there are some uncertainties associated with the QUADAS-2 evaluation, the risks of uncertainty may not significantly affect the results and therefore the overall quality of the study can be analyzed.

## Conclusion

In conclusion, radiomics is a useful tool for predicting LMN in patients with BTCs. Radiomics study on LMN prediction, however, is still in its early phases. Further study on the quality of radiomics is required in the segmentation of ROI, method repeatability, model building, and overfitting solutions. To demonstrate the clinical value of radiomics, further high-quality, multicenter, large-scale prospective trials are required.

## Data Availability

The original contributions presented in the study are included in the article/**Supplementary Material**, further inquiries can be directed to the corresponding author/s.

## References

[1] Mavros MN Economopoulos KP Alexiou VG Pawlik TM. Treatment and prognosis for patients with intrahepatic cholangiocarcinoma: systematic review and meta-analysis. JAMA Surg. (2014) 149:565–74. 10.1001/jamasurg.2013.5137 24718873

[2] Hundal R Shaffer EA. Gallbladder cancer: epidemiology and outcome. Clin Epidemiol. (2014) 6:99–109. 10.2147/CLEP.S37357 24634588 PMC3952897

[3] Khan SA Clements O Jin UK Joseph E. Reply to: “letter regarding [risk factors for intrahepatic and extrahepatic cholangiocarcinoma: a systematic review and meta-analysis]”. J Hepatol. (2020) 72:95–103. 10.1016/S0168-8278(20)30719-4 31536748

[4] DeSantis CE Kramer JL Jemal A. The burden of rare cancers in the United States. CA: a Cancer J Clin. (2017) 67:261–72. 10.3322/caac.21400 28542893

[5] Horgan AM Amir E Walter T Knox JJ. Adjuvant therapy in the treatment of biliary tract cancer: a systematic review and meta-analysis. J Clin Oncol. (2012) 30:1934–40. 10.1200/JCO.2011.40.5381 22529261

[6] Chan E Berlin J. Biliary tract cancers: understudied and poorly understood. J Clin Oncol. (2015) 33:1845–8. 10.1200/JCO.2014.59.7591 25918294

[7] Razumilava N Gores GJ. Cholangiocarcinoma. Lancet (London, England). (2014) 383:2168–79. 10.1016/S0140-6736(13)61903-0 24581682 PMC4069226

[8] Zhang XF Beal EW Bagante F Chakedis J Weiss M Popescu I Early versus late recurrence of intrahepatic cholangiocarcinoma after resection with curative intent. Br J Surg. (2018) 105:848–56. 10.1002/bjs.10676 29193010

[9] Mao ZY Guo XC Su D Wang LJ Zhang TT Bai L. Prognostic factors of cholangiocarcinoma after surgical resection: a retrospective study of 293 patients. Med Sci Moni. (2015) 21:2375–81. 10.12659/MSM.893586 PMC4540057 26269932

[10] Rizvi S Khan SA Hallemeier CL Kelley RK Gores GJ. Cholangiocarcinoma - evolving concepts and therapeutic strategies. Nat Rev Clin Oncol. (2018) 15:95–111. 10.1038/nrclinonc.2017.157 28994423 PMC5819599

[11] Wang Y Zhou CW Zhu GQ Li N Qian XL Chong HH A multidimensional nomogram combining imaging features and clinical factors to predict the invasiveness and metastasis of combined hepatocellular cholangiocarcinoma. Ann Transl Med. (2021) 9:1518. 10.21037/atm-21-2500 34790724 PMC8576707

[12] Zhou Y Zhou G Gao X Xu C Wang X Xu P. Apparent diffusion coefficient value of mass-forming intrahepatic cholangiocarcinoma: a potential imaging biomarker for prediction of lymph node metastasis. Abdom Radiol (New York). (2020) 45:3109–18. 10.1007/s00261-020-02458-x 32107582

[13] Saleh M Virarkar M Bura V Valenzuela R Javadi S Szklaruk J Intrahepatic cholangiocarcinoma: pathogenesis, current staging, and radiological findings. Abdom Radiol (New York). (2020) 45:3662–80. 10.1007/s00261-020-02559-7 32417933

[14] Blechacz B Komuta M Roskams T Gores GJ. Clinical diagnosis and staging of cholangiocarcinoma. Nature Reviews Gastroenterology & Hepatol. (2011) 8:512–22. 10.1038/nrgastro.2011.131 PMC3331791 21808282

[15] Huang YQ Liang CH He L Tian J Liang CS Chen X Development and validation of a radiomics nomogram for preoperative prediction of lymph node metastasis in colorectal cancer. J Clin Oncol. (2016) 34:2157–64. 10.1200/JCO.2015.65.9128 27138577

[16] Liang W Xu L Yang P Zhang L Wan D Huang Q Novel nomogram for preoperative prediction of early recurrence in intrahepatic cholangiocarcinoma. Front Oncol. (2018) 8:360. 10.3389/fonc.2018.00360 30234019 PMC6131601

[17] Tang Y Zhang T Zhou X Zhao Y Xu H Liu Y The preoperative prognostic value of the radiomics nomogram based on CT combined with machine learning in patients with intrahepatic cholangiocarcinoma. World J Surg Oncol. (2021) 19:45. 10.1186/s12957-021-02162-0 34334138 PMC8327418

[18] Ji GW Zhu FP Zhang YD Liu XS Wu FY Wang K A radiomics approach to predict lymph node metastasis and clinical outcome of intrahepatic cholangiocarcinoma. Eur Radiol. (2019) 29:3725–35. 10.1007/s00330-019-06142-7 30915561

[19] Yang C Huang M Li S Chen J Yang Y Qin N Radiomics model of magnetic resonance imaging for predicting pathological grading and lymph node metastases of extrahepatic cholangiocarcinoma. Cancer Lett. (2020) 470:1–7. 10.1016/j.canlet.2019.11.036 31809800

[20] Tang Y Yang CM Su S Wang WJ Fan LP Shu J. Machine learning-based radiomics analysis for differentiation degree and lymphatic node metastasis of extrahepatic cholangiocarcinoma. BMC cancer. (2021) 21:1268. 10.1186/s12885-021-08947-6 34819043 PMC8611922

[21] McInnes MDF Moher D Thombs BD McGrath TA Bossuyt PM Clifford T Preferred reporting items for a systematic review and meta-analysis of diagnostic test accuracy studies: the PRISMA-DTA statement. Jama. (2018) 319:388–96. 10.1001/jama.2017.19163 29362800

[22] Huang J Tian W Zhang L Huang Q Lin S Ding Y Preoperative prediction power of imaging methods for microvascular invasion in hepatocellular carcinoma: a systemic review and meta-analysis. Front Oncol. (2020) 10:887. 10.3389/fonc.2020.00887 32676450 PMC7333535

[23] Lambin P Leijenaar RTH Deist TM Peerlings J de Jong EEC van Timmeren J Radiomics: the bridge between medical imaging and personalized medicine. Nat Rev Clin Oncol. (2017) 14:749–62. 10.1038/nrclinonc.2017.141 28975929

[24] Moses LE Shapiro D Littenberg B. Combining independent studies of a diagnostic test into a summary ROC curve: data-analytic approaches and some additional considerations. Stat Med. (1993) 12:1293–316. 10.1002/sim.4780121403 8210827

[25] Higgins JP Thompson SG Deeks JJ Altman DG. Measuring inconsistency in meta-analyses. BMJ (Clin Res ed). (2003) 327:557–60. 10.1136/bmj.327.7414.557 PMC192859 12958120

[26] Deeks JJ Macaskill P Irwig L. The performance of tests of publication bias and other sample size effects in systematic reviews of diagnostic test accuracy was assessed. J Clin Epidemiol. (2005) 58:882–93. 10.1016/j.jclinepi.2005.01.016 16085191

[27] Hellmich M Lehmacher W. A ruler for interpreting diagnostic test results. Methods Inf Med. (2005) 44:124–6. 10.1055/s-0038-1633930 15778803

[28] Ji GW Zhang YD Zhang H Zhu FP Wang K Xia YX Biliary tract cancer at CT: a radiomics-based model to predict lymph node metastasis and survival outcomes. Radiol. (2019) 290:90–8. 10.1148/radiol.2018181408 30325283

[29] Liu X Liang X Ruan L Yan S. A clinical-radiomics nomogram for preoperative prediction of lymph node metastasis in gallbladder cancer. Front Oncol. (2021) 11:633852. 10.3389/fonc.2021.633852 34631512 PMC8493033

[30] Xu L Yang P Liang W Liu W Wang W Luo C A radiomics approach based on support vector machine using MR images for preoperative lymph node status evaluation in intrahepatic cholangiocarcinoma. Theranostics. (2019) 9:5374–85. 10.7150/thno.34149 31410221 PMC6691572

[31] Yao X Huang X Yang C Hu A Zhou G Lei J A novel approach to assessing differentiation degree and lymph node metastasis of extrahepatic cholangiocarcinoma: prediction using a radiomics-based particle swarm optimization and support vector machine model. JMIR Med Inform. (2020) 8:e23578. 10.2196/23578 33016889 PMC7573697

[32] Huang C. Development and validation of a preoperative radiomics nomogram for prediction of lymph node metastasis of intrahepatice cholangiocarcinoma.: Shanghai, China: Naval Medical University; 2019.

[33] Bridgewater J Galle PR Khan SA Llovet JM Park JW Patel T Guidelines for the diagnosis and management of intrahepatic cholangiocarcinoma. J Hepatol. (2014) 60:1268–89. 10.1016/j.jhep.2014.01.021 24681130

[34] Yu Y He Z Ouyang J Tan Y Chen Y Gu Y Magnetic resonance imaging radiomics predicts preoperative axillary lymph node metastasis to support surgical decisions and is associated with tumor microenvironment in invasive breast cancer: a machine learning, multicenter study. EBioMedicine. (2021) 69:103460. 10.1016/j.ebiom.2021.103460 34233259 PMC8261009

[35] Mayerhoefer ME Materka A Langs G Häggström I Szczypiński P Gibbs P Introduction to radiomics. J Nuc Med. (2020) 61:488–95. 10.2967/jnumed.118.222893 PMC9374044 32060219

[36] Wang Y Shao J Wang P Chen L Ying M Chai S Deep learning radiomics to predict regional lymph node staging for hilar cholangiocarcinoma. Front Oncol. (2021) 11:721460. 10.3389/fonc.2021.721460 34765542 PMC8576333

[37] Rizzo S Botta F Raimondi S Origgi D Fanciullo C Morganti AG Radiomics: the facts and the challenges of image analysis. Euro Radiol Exp. (2018) 2:36. 10.1186/s41747-018-0068-z PMC6234198 30426318

[38] Lafata KJ Wang Y Konkel B Yin FF Bashir MR. Radiomics: a primer on high-throughput image phenotyping. Abdominal Radiol (New York). (2022) 47:2986–3002. 10.1007/s00261-021-03254-x. 34435228

[39] Hwang J Kim YK Park MJ Lee MH Kim SH Lee WJ Differentiating combined hepatocellular and cholangiocarcinoma from mass-forming intrahepatic cholangiocarcinoma using gadoxetic acid-enhanced MRI. J Magn Res Imag: JMRI. (2012) 36:881–9. 10.1002/jmri.23728 22730271

[40] Park TG Yu YD Park BJ Cheon GJ Oh SY Kim DS Implication of lymph node metastasis detected on 18F-FDG PET/CT for surgical planning in patients with peripheral intrahepatic cholangiocarcinoma. Clin Nucl Med. (2014) 39:1–7. 10.1097/RLU.0b013e3182867b99 24335565

