# Supplementary material for: A meta-analysis of based radiomics for predicting lymph node metastasis in patients with biliary tract cancers
Source: Front Surg. 2023 Jan 6;9:1045295. doi: 10.3389/fsurg.2022.1045295 (PMC9852536; doi:10.3389/fsurg.2022.1045295)
Supplement: Supplementary file 1 [file Table1.docx]

Table S1. The RQS for author 1.

| **Study Criteria** | **Ji**  **2019** | **Yao**  **2020** | **Xu**  **2019** | **Liu**  **2021** | **Ji**  **2018** | **Yang**  **2020** | **Huang**  **2019** |
| --- | --- | --- | --- | --- | --- | --- | --- |
| Image protocol quality | +2 | +2 | +2 | +2 | +2 | +2 | +2 |
| Multiple segmentations | +1 | +0 | +1 | +1 | +1 | +1 | +0 |
| Phantom study on all scanners | +0 | +0 | +0 | +0 | +0 | +0 | +0 |
| Imaging at multiple time points | +0 | +0 | +0 | +0 | +0 | +0 | +0 |
| Feature reduction or adjustment for multiple testing | +3 | +3 | +3 | +3 | +3 | +3 | +3 |
| Multivariable analysis with non-radiomics feature | +1 | +0 | +1 | +1 | +1 | +0 | +1 |
| Detect and discuss biological correlates | +0 | +0 | +0 | +0 | +0 | +0 | +0 |
| Cutoff analyses | +1 | +0 | +0 | +0 | +1 | +1 | +1 |
| Discrimination statistics | +1 | +2 | +1 | +2 | +1 | +2 | +1 |
| Calibration statistic | +1 | +0 | +2 | +2 | +2 | +1 | +1 |
| Prospective study registered in a trial database | +0 | +0 | +0 | +0 | +0 | +0 | +0 |
| Validation | +2 | +2 | +2 | +3 | +2 | +2 | +2 |
| Comparison to “gold standard” | +2 | +0 | +2 | +2 | +2 | +0 | +2 |
| Potential clinical utility | +2 | +0 | +2 | +2 | +2 | +0 | +0 |
| Cost-effectiveness analysis | +0 | +0 | +0 | +0 | +0 | +0 | +0 |
| Open science and data | +2 | +2 | +2 | +2 | +2 | +3 | +2 |
| Total score (Maximum:36) | 18 | 11 | 18 | 20 | 19 | 15 | 15 |
| Individual Rating | 50.0％ | 30.5％ | 50.0％ | 56％ | 52.8％ | 41.7％ | 41.7％ |

Table S2. The RQS for author 2.

| **Study Criteria** | **Ji**  **2019** | **Yao**  **2020** | **Xu**  **2019** | **Liu**  **2021** | **Ji**  **2018** | **Yang**  **2020** | **Huang**  **2019** |
| --- | --- | --- | --- | --- | --- | --- | --- |
| Image protocol quality | +2 | +2 | +2 | +2 | +2 | +2 | +2 |
| Multiple segmentations | +0 | +0 | +1 | +1 | +1 | +1 | +0 |
| Phantom study on all scanners | +0 | +0 | +0 | +0 | +0 | +0 | +0 |
| Imaging at multiple time points | +0 | +0 | +0 | +0 | +0 | +0 | +0 |
| Feature reduction or adjustment for multiple testing | +3 | +3 | +3 | +3 | +3 | +3 | +3 |
| Multivariable analysis with non-radiomics feature | +1 | +0 | +1 | +1 | +1 | +0 | +1 |
| Detect and discuss biological correlates | +0 | +0 | +0 | +0 | +0 | +0 | +0 |
| Cutoff analyses | +1 | +0 | +0 | +0 | +1 | +1 | +1 |
| Discrimination statistics | +1 | +2 | +1 | +2 | +2 | +1 | +2 |
| Calibration statistic | +1 | +1 | +1 | +1 | +2 | +1 | +1 |
| Prospective study registered in a trial database | +0 | +0 | +0 | +0 | +0 | +0 | +0 |
| Validation | +2 | +2 | +2 | +3 | +2 | +2 | +2 |
| Comparison to “gold standard” | +2 | +0 | +2 | +2 | +2 | +0 | +2 |
| Potential clinical utility | +2 | +0 | +2 | +2 | +2 | +0 | +0 |
| Cost-effectiveness analysis | +0 | +0 | +0 | +0 | +0 | +0 | +0 |
| Open science and data | +2 | +2 | +2 | +2 | +2 | +3 | +2 |
| Total score (Maximum:36) | 17 | 12 | 17 | 19 | 20 | 14 | 16 |
| Individual Rating | 47.2 | 33.3 | 47.2 | 52.8 | 55.6 | 38.9 | 44.4 |
